# LPS-Induced Lung Inflammation in Marmoset Monkeys – An Acute Model for Anti-Inflammatory Drug Testing

**DOI:** 10.1371/journal.pone.0043709

**Published:** 2012-08-28

**Authors:** Sophie Seehase, Hans-Dieter Lauenstein, Christina Schlumbohm, Simone Switalla, Vanessa Neuhaus, Christine Förster, Hans-Gerd Fieguth, Olaf Pfennig, Eberhard Fuchs, Franz-Josef Kaup, Martina Bleyer, Jens M. Hohlfeld, Armin Braun, Katherina Sewald, Sascha Knauf

**Affiliations:** 1 Airway Research, Fraunhofer Institute for Toxicology and Experimental Medicine, Hannover, Germany; 2 Pathology Unit, German Primate Center, Leibniz-Institute for Primate Research, Göttingen, Germany; 3 Encepharm GmbH, Göttingen, Germany; 4 Institute of Pathology, Klinikum Region Hannover Klinikum Nordstadt, Hannover, Germany; 5 Division of Thoracic Surgery, Klinikum Region Hannover Klinikum Oststadt-Heidehaus, Hannover, Germany; University Hospital Freiburg, Germany

## Abstract

Increasing incidence and substantial morbidity and mortality of respiratory diseases requires the development of new human-specific anti-inflammatory and disease-modifying therapeutics. Therefore, new predictive animal models that closely reflect human lung pathology are needed. In the current study, a tiered acute lipopolysaccharide (LPS)-induced inflammation model was established in marmoset monkeys (*Callithrix jacchus)* to reflect crucial features of inflammatory lung diseases. Firstly, in an *ex vivo* approach marmoset and, for the purposes of comparison, human precision-cut lung slices (PCLS) were stimulated with LPS in the presence or absence of the phosphodiesterase-4 (PDE4) inhibitor roflumilast. Pro-inflammatory cytokines including tumor necrosis factor-alpha (TNF-α) and macrophage inflammatory protein-1 beta (MIP-1β) were measured. The corticosteroid dexamethasone was used as treatment control. Secondly, in an *in vivo* approach marmosets were pre-treated with roflumilast or dexamethasone and unilaterally challenged with LPS. Ipsilateral bronchoalveolar lavage (BAL) was conducted 18 hours after LPS challenge. BAL fluid was processed and analyzed for neutrophils, TNF-α, and MIP-1β. TNF-α release in marmoset PCLS correlated significantly with human PCLS. Roflumilast treatment significantly reduced TNF-α secretion *ex vivo* in both species, with comparable half maximal inhibitory concentration (IC_50_). LPS instillation into marmoset lungs caused a profound inflammation as shown by neutrophilic influx and increased TNF-α and MIP-1β levels in BAL fluid. This inflammatory response was significantly suppressed by roflumilast and dexamethasone. The close similarity of marmoset and human lungs regarding LPS-induced inflammation and the significant anti-inflammatory effect of approved pharmaceuticals assess the suitability of marmoset monkeys to serve as a promising model for studying anti-inflammatory drugs.

## Introduction

Inflammatory lung diseases including pneumonia, acute lung injury (ALI), acute respiratory distress syndrome (ARDS), and chronic obstructive pulmonary disease (COPD) cause significant morbidity and mortality worldwide and display a major public health impact [1]; [2]. On cellular level, these respiratory diseases are based on inflammation which can be either acute or chronic. The inflammatory process is characterized by an increased expression of multiple cytokines and chemokines. In particular, activated macrophages and epithelial cells produce inflammatory mediators such as tumor necrosis factor alpha (TNF-α) and interleukin-1 beta (IL-1β) which in turn induce the attraction of neutrophils and the release of further cytokines including IL-6 [3]. These inflammatory aspects of cytokine up-regulation can also be mimicked in *in-vitro, ex vivo,* as well as *in vivo* approaches by using infectious or environmental stimuli [4]–[8]. Especially the endotoxin lipopolysaccharide (LPS), which is part of the outer membrane of gram-negative bacteria, is one of the most potent immune-activating stimuli known. LPS induces a profound activation of the innate immunity via CD14 and Toll-like receptor (TLR) 4 that results in a strong inflammatory response due to activation of the transcription factor NF-κΒ [9]; [10]. LPS is, therefore, widely used to model features of inflammatory diseases *in vitro* as well as *in vivo*.

Acute respiratory LPS challenge models in animals as well as in humans which are characterized by bronchoalveolar neutrophil influx and cytokine up-regulation, have extensively been used for the testing of new anti-inflammatory drugs [4]; [8]; [11]; [12], although they do not reflect all features of human, notably chronic, respiratory diseases. So far, corticosteroids have widely been used for the anti-inflammatory treatment of inflammatory lung diseases. However, they provide only little benefit in disease progression or mortality [13]; [14]. Thus, development of new, effective anti-inflammatory drugs is urgently needed. Particularly, antagonists or inhibitors targeting the mechanisms involved in recruitment and accumulation of inflammatory cells, including neutrophils, display promising options for therapeutic intervention in lung inflammation [15]. In fact, a first success has been achieved with the highly potent phosphodiesterase-4 (PDE4) inhibitor roflumilast [11]; [16], which was extensively tested on acute respiratory LPS challenge models [4]; [8]; [11].

Preclinical testing of highly specific anti-inflammatory drugs requires valid translational animal models [17]. Yet, rodent models have commonly been used, even though they are often limited in reflecting the human pathology [18]; [19]. Rodents in contrast to non-human primates (NHP) or humans have less extensive airway branching and normally do not have respiratory bronchioles [19]. The close phylogenetic relationship between NHP and humans, and the resulting high homology to a variety of human target structures make NHP interesting for preclinical testing of newly developed drugs [18]. The New World monkey common marmoset (*Callithrix jacchus*), for instance, is widely used as a model of human inflammatory diseases of the central nervous system, such as experimental autoimmune encephalomyelitis (EAE) to model multiple sclerosis [20]; [21]. Because of its phylogenetic proximity to humans and its small body size of about 400 grams the marmoset monkey seems to be of high value for modeling human respiratory diseases. Furthermore, marmoset monkeys have already been used for inhalation studies [22]; [23].

The rationale of the present study was to establish a tired translational LPS-induced acute lung inflammation model in the marmoset monkey for preclinical testing of anti-inflammatory drugs. Both, an *ex vivo* and *in vivo* approach of LPS-induced acute inflammation were used to reflect inflammatory lung diseases. Firstly, we investigated whether marmoset precision-cut lung slices (PCLS) display a similar inflammatory response upon LPS exposure as seen in human PCLS studies [7]. Secondly, we analyzed the effect of an acute unilateral LPS challenge in marmoset monkeys. The study was designed close to a clinical trial conducted by our Clinical Airway Research department, where segmental LPS challenge was performed in healthy subjects after roflumilast treatment [11]. By using the PDE4 inhibitor roflumilast and for control the corticosteroid dexamethasone we investigated the therapeutic efficacy of immunosuppressive drugs *ex vivo* and *in vivo* against the acute LPS-induced inflammatory response.

## Materials and Methods

### Animals

Experiments were performed in adult common marmoset monkeys (*Callithrix jacchus*) at the German Primate Center, Goettingen, Germany. Animal care and housing conditions complied with the regulations of the European Parliament and the European Council Directive on the protection of animals used for scientific purposes (2010/63/EU) and also with the National Institutes of Health Guide for the Care and Use of Laboratory Animals. Briefly, animals were housed in pairs in standardized and commercially available wire mesh cages (cage size 80×65×150 cm, Bioscape, Castrop-Rauxel, Germany). In each cage a sleeping box and wooden sitting boards were installed. The floor under the cages was covered by paper sheets. Urine and faeces were removed daily by exchange of the paper sheets. Moreover, the cages were cleaned in weekly intervals and disinfected with water and Biguacid (Antiseptica, Polheim/Brauweiler, Austria). Room temperature was maintained at 26±1.5°C and the relative humidity was kept between 60 and 80%. Artificial light was set to give a cycle of 12 hours light and 12 hours darkness. Room air was changed approximately 8 times per hour and filtered adequately. All materials were changed regularly, cleaned and sterilized. In order to define hygiene status of the colony and to exclude infection with enteral pathogens, faecal samples were collected from the animal housing rooms for bacteriological analysis every three months. Animals received water and a pelletized marmoset diet (ssniff® Spezialdiäten GmbH, Soest, Germany) *ad libitum*. Water quality was controlled on a regular basis.

The experiments were approved by the Lower Saxony Federal State Office for Consumer Protection and Food Safety, Germany (reference number AZ 33.11.42502-04-10/0032). All procedures, except oral administration of dexamethasone or roflumilast or sham, and blood withdrawal, were performed in anesthetized animals. Anesthesia was performed using a combination of Alfaxan® (Vétoquinol, Ravensburg, Germany; 12–18 mg alfaxalon/kg i.m.), Diazepam® (Ratiopharm, Ulm, Germany; 0.5–1.5 mg diazepam/kg i.m.), and Robinul® (Riemser, Greifswald, Germany; 0.02–0.05 mg glycopyrronium bromide/kg s.c.) [24].

Animals used for *ex vivo* experiments were euthanized during general anesthesia with sodium pentobarbital (Narcoren®, Merial GmbH, Hallbergmoos, Germany; 400 mg kg/bw i.v.) according to EU Guideline 2010/63/EU. Table 1 shows the animals used for the *in vivo* experiments. Lungs for *ex vivo* studies were used from animals with an average age of 6±2 years. All of them were part of control groups and were not pre-treated with any substances.

**Table 1:** Demographic data of the study population.

	sham (n = 8)	dxm (n = 7)	rof (n = 7)
**Median age [years]**	4.0±0.6	3.9±0.3	4.4±0.1
**Body weight [g]**	440±51	409±25	429±57
**Sex, n**			
**Male**	4	3	2
**Female**	4	4	5
**Animal number**	#13901 ^a^	#13958 ^a^	#13859^ a^
	#14374 ^a^	#13763^ a^	#13866^ a^
	#13952 ^a^	#13853^ a^	#13605^ a^
	#14168 ^a^	#14168^ b^	#13763^ b^
	#14299 ^a^	#13866^ b^	#13863^ b^
	#13859^ b^	#13853^ b^	#13958^ b^
	#14374^ b^	#12785^ b^	#11595^ b^
	#13564^ b^		
*Excluded*	*#13605^ b^*	*#13863^ a^*	*#13526^ a^*

Animals were randomized in each of the two independent study cycles as indicated (a: first study cycle, b: second study cycle). Before each cycle a baseline BAL was performed 3 weeks before LPS challenge and served as control. Altogether, 3 animals had to be excluded.

Data are given as mean ± S.E.M., dxm  =  dexamethasone, rof  =  roflumilast.

### Culture Media and Reagents

Dulbecco's Modified Eagle's Medium Nutrient Mixture F-12 Ham (DMEM) with L-glutamine, 15 mM HEPES, pH 7.2–7.4 without phenol red and foetal calf serum was complemented with 7.5% (w/v) sodium bicarbonate (all Sigma Aldrich, München, Germany). DMEM and RPMI 1640 (Lonza, Verviers, Belgium) were supplemented with 100 U/mL penicillin and 100 µg/mL streptomycin (Sigma Aldrich). LPS (*E. coli*, serotype 0111:B4) was supplied in lyophilized form by Sigma Aldrich and dissolved in PBS (Lonza), pH 7.4.

### Human Donors

The experiments performed with human tissue were approved by the ethics committee of the Hannover Medical School. Patients gave written informed consent. Human lung lobes were obtained from patients who underwent lobectomy because of cancer. Only tumor-free lung tissue was used. Tissue was processed immediately on the day of resection. Table 2 characterizes the human lung tissue donors. The average age of patients was 60±10 years (male and female), and 80% of them were smokers.

**Table 2:** Characterization of human lung tissue donors.

Donor	Age	Sex	Location	Smokestatus	Classification	Cancer Type
# 1	66	Female	Left inferior lobe	–	–	Adenocarcinoma
#2	63	Male	Right inferior lobe	20 py	GOLD II	Basaloid squamous cell carcinoma
#3	65	Female	Right inferior lobe	50 py	GOLD I	Squamous cell carcinoma
#4	65	Male	Medial lobe	40 py	GOLD II	Adenocarcinoma

py  =  pack-years of smoking.

### Whole-blood Assay (WBA)

Marmoset heparinised whole-blood was obtained from the German Primate Center. Whole-blood was incubated with RPMI (diluted to 1∶10) containing the indicated concentrations of LPS under standard cell culture conditions (37°C, 5% CO_2_, and 95% humidified atmosphere). Dexamethasone (Ratiopharm, Ulm, Germany) was used for positive treatment control. Whole-blood cultures without addition of substances served as reference. After 24 hours of incubation, whole-blood cultures were centrifuged. Whole-blood culture supernatants were subsequently collected, supplemented with 0.2% protease inhibitor cocktail (Sigma Aldrich), and stored at −80°C for further analysis.

### Preparation of Precision-cut Lung Slices (PCLS)

Human and marmoset PCLS were prepared as previously described [7]; [25]. Briefly, lungs or lung lobes were filled with warm (37°C), liquid, low gelling temperature agarose (1.5% (w/v), Sigma Aldrich) in DMEM. The agarose-filled tissue was allowed to solidify on ice. Tissue cores were prepared with a rotating sharpened metal tube (diameter 8 mm) and cut into sections of approximately 250 µm with a microtome (Krumdieck tissue slicer, Alabama Research and Development, Munford, AL, USA) in Earle's Balanced Salt Solution (Sigma Aldrich). Thus, approximately 300 PCLS per specimen were produced. PCLS were incubated in DMEM containing different concentrations of LPS (2.5 to 500 ng/mL LPS) under standard cell culture conditions. Additionally, PCLS were incubated with 0.6 to 310 nM roflumilast (Daxas®, Nycomed, Konstanz, Germany) and 100 ng/mL LPS to determine the half maximal inhibitory concentration (IC_50_) of roflumilast. Untreated PCLS were used as reference, dexamethasone-treated PCLS (50 µg/mL) served as treatment control. Approaches were performed in duplicates containing four PCLS of one specimen. Supernatants were collected after 24 hours of incubation, supplemented with 0.2% protease inhibitor cocktail, and stored at −80°C until further analysis. Moreover, PCLS were lysed with 1% Triton X-100 in PBS (1 h, 4°C) for measurement of intracellular cytokine levels. For the purpose of standardization the cytokine amount per approach was calculated in relation to the protein content.

### Study Design of in vivo LPS Stimulation

**Figure 1:**
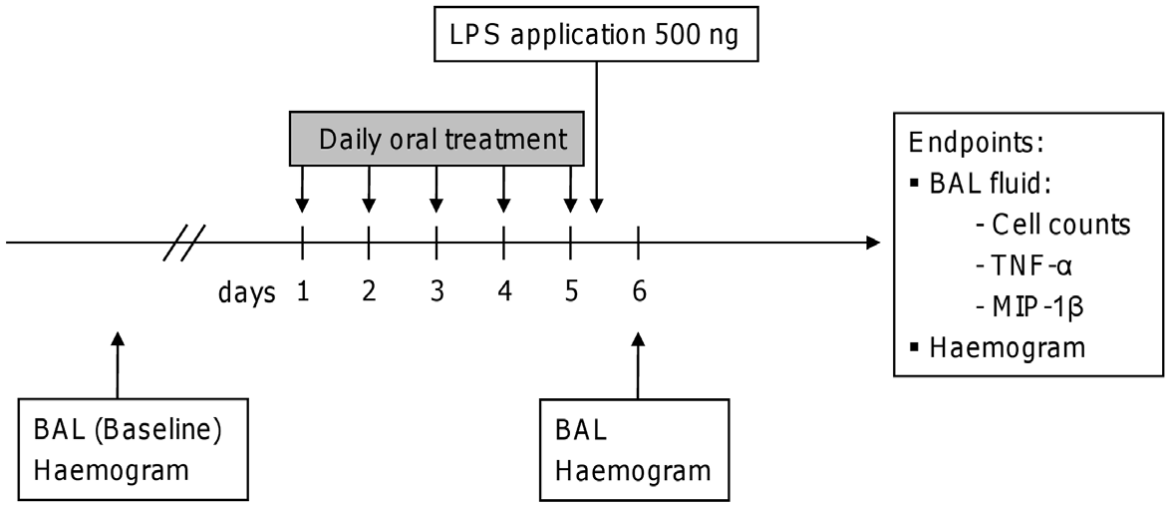
Study design of LPS-induced lung inflammation in marmoset monkeys. Animals were stimulated intrabronchially with 500 ng LPS instilled unilaterally after 5-day oral pre-treatment with either roflumilast (7 µg/kg bw) or dexamethasone (2 mg/kg bw). Sham-treated individuals receiving water were used as positive controls. Unilateral bronchoalveolar lavage (BAL) was performed 18 hours after LPS provocation. BAL fluid was analyzed for neutrophils, TNF-α and MIP-1β secretion. Baseline levels were determined three weeks before the first treatment and served as control.

Marmosets were health-screened in advance to study-start. Screening included an initial haemogram, blood chemistry, and bronchoalveolar lavage (BAL). The initial BAL served as an individual baseline control for each animal. After a recovery time of at least three weeks, animals were orally pre-treated with roflumilast (7 µg/kg bw) on five consecutive days according to Figure 1. Additionally, pre-treatment with dexamethasone (2 mg/kg bw) served as treatment control, while animals receiving water served as positive control (sham). Roflumilast or dexamethasone were dissolved in 300 µL water and mixed with 700 µL of a nutritional substitution (Nutri-Cal®, Albrecht, Aulendorf, Germany) to encourage unsolicited uptake by the animals. On day 5, animals were anaesthetized and received unilateral LPS administration, after a bronchoscope had been placed into the left bronchus under video control. A MicroSprayer® (PennCentury, Philadelphia, PA, USA) was inserted through the working channel of the bronchoscope and an aerosol of 100 µL saline (0.9% NaCl, Braun, Melsungen, Germany) containing a total dose of 500 ng LPS was administered. Eighteen hours later, animals were anaesthetized again and underwent another bronchoscopy for collection of BAL fluid from the challenged lung lobe.

BAL fluid was processed and analyzed for total and differential cell counts as well as cytokine levels. A third BAL was performed after a minimum of three weeks post LPS instillation to exclude long-term effects. The study was performed twice with an interval of 12 months to check reproducibility and aiming to reduce the number of animals. In each study cycle a baseline BAL representing baseline control was performed 3 weeks before LPS challenge. Furthermore, a power analysis was used to enhance the validity of the study. Animals in both cycles were randomized enabling the pooled analysis of both study cycles (Tab. 1).

### Bronchoscopic Procedure for BAL Sample Retrieval

Marmosets were food and water deprived for at least three hours prior to anesthesia. Animals were anesthetized as described above and placed into dorsal recumbency. The animal’s head was slightly tilted towards the neck. Body temperature was maintained at 37°C utilizing a heating pad. The mouth was opened with a 75-mm laryngoscope spatula (Heine Classic+® Miller 00 F.O., Herrsching, Germany), while larynx and trachea were brought into a horizontal line. Medical-silicon spray (Servoprax, Wesel, Germany) was carefully used to lubricate the distal part of the endoscope. The custom-made bronchoscope (OD 2.5 mm, length 250 mm, Karlheinz Hinze Optoengineering, Hamburg, Germany) was inserted into the trachea and placed into the *bronchus principalis sinister*. The left lung was flushed twice with 3 mL of warm saline solution. Finally, the bronchoscope was gently removed from the trachea. Oxygen saturation and heart rate were permanently monitored with a pulse oxymeter (Medair, Hudiksvall, Sweden). For the purpose of standardization, only the second BAL fluid was obtained. Additionally, a recovery volume of more than 2 mL was required.

### Analysis of Tissue Culture Supernatants and BAL

Concentrations of TNF-α were quantified by Enzyme Linked Immunosorbent Assay (ELISA; New World monkey TNF-α ELISA, U-Cy-Tech, Utrecht, the Netherlands; human TNF-α DuoSet, R&D Systems, Wiesbaden-Nordenstadt, Germany) with a lower limit of detection of 5 pg/mL. Macrophage inflammatory protein-1beta (MIP-1β) was measured using Luminex technology (Millipore, Schwalbach, Germany) with a lower limit of quantification of 0.64 pg/mL. Total protein concentrations of PCLS were determined with the Bicinchoininc acid (BCA) method using a commercial kit according to the manufacturer’s instructions (BCA, Protein Assay Kit, Pierce, Rockford, IL, USA). Total cell counts in BAL fluid were determined using a Scepter™ cell counter (Millipore, Schwalbach, Germany). Differential cell counts of 400 cells per slide were performed visually after Pappenheim staining.

### Statistics

Data are expressed as mean ± SEM or median, as indicated in the figures. By using GraphPad Prism 4.0 (GraphPad, San Diego, CA, USA) a one-tailed Mann-Whitney U-test was performed to answer the question, whether roflumilast treatment reduces LPS-induced inflammation. A Grubbs’ test with a significance level of 0.05 was performed to detect outliers. The number of subjects is indicated in the figure legends. Differences between treated samples and controls were considered statistically significant at a level of p<0.05. Correlations were evaluated using a linear regression analysis model combined with Spearman’s rank correlation coefficient (r_s_). Dose-response curves were created using a nonlinear regression model of normalized values with variable slope.

## Results

### LPS Triggers an Increase in Pro-inflammatory Mediators in Marmoset Monkeys Ex Vivo – Correlation with Human Data

**Figure 2:**
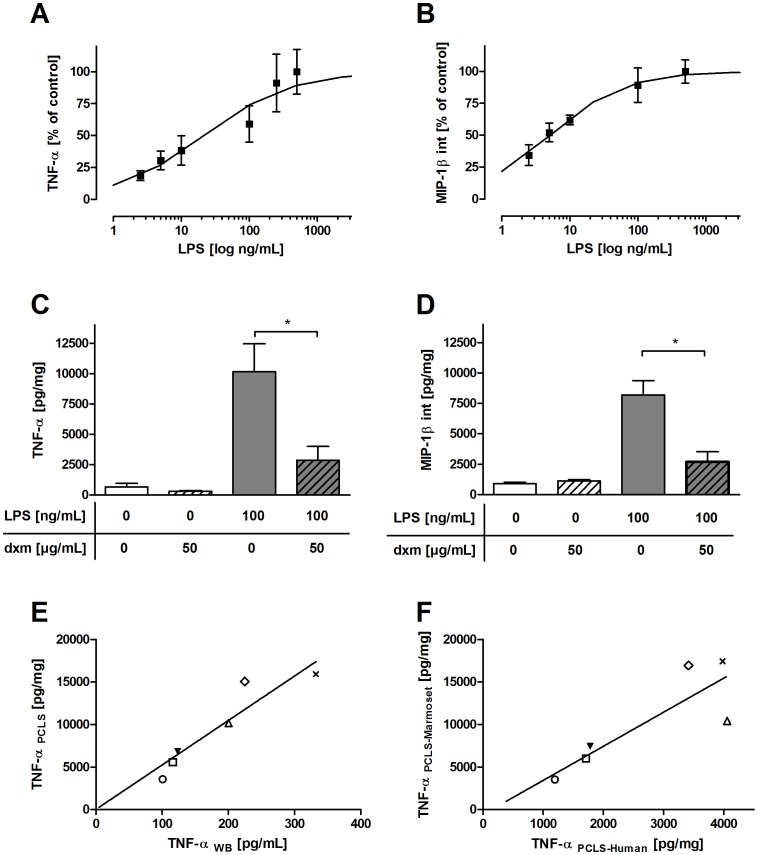
LPS-dependent increase in cytokines and chemokines *ex vivo*. Ascending TNF-α (A) and intracellular MIP-1β (B) production after 24-hour incubation with increasing concentrations of LPS in marmoset PCLS. LPS-induced increase in TNF-α (C) and intracellular MIP-1β (D) in marmoset PCLS was significantly suppressed by dexamethasone (dxm). Marmoset PCLS and marmoset WBA (E) on the one hand and marmoset PCLS and human PCLS (F) on the other hand showed significant correlations for TNF-α secretion (Spearman’s rank correlation coefficient (r_S_)  = 1.0 with p  = 0.0004, and r_s_  = 0.9 with p  = 0.01, respectively). Symbols: ○: 2.5 ng/mL LPS, □: 5 ng/mL LPS, ▾: 10 ng/mL LPS, ▵: 100 ng/mL LPS, ◊: 250 ng/mL LPS, x: 500 ng/mL LPS. Data are presented as mean ± SEM, *p<0.05, **p<0.01, Mann-Whitney test (TNF-α: n  = 6, MIP-1β: n  = 4). Correlations were evaluated using a linear regression analysis model combined with Spearman’s rank correlation coefficient. NHP  =  non-human primate, int  =  intracellular.

The ability of marmoset monkeys to respond to the well-established immunomodulator LPS was first tested *ex vivo* in whole-blood cultures and vital lung tissue. Marmoset whole blood and PCLS were exposed to LPS, and the effect on cytokine release was determined. The LPS-induced acute inflammatory response in both blood cultures and vital lung tissue was characterized by rapid accumulation of pro-inflammatory cytokines such as TNF-α and MIP-1β. LPS significantly increased the release of TNF-α (control: 670 pg/mL vs. 500 ng/mL LPS: 16,700 pg/mL) and intracellular production of MIP-1β (control: 900 pg/mL vs. 500 ng/mL LPS: 12,600 pg/mL) in marmoset lung tissue (Fig. 2A, B). The half maximal effective concentration (EC_50_) was 22 ng/mL LPS for TNF-α and 5 ng/mL LPS for intracellular MIP-1β. Treatment with dexamethasone reduced LPS-elicited cytokine levels of TNF-α to 72% and of MIP-1β to 67% (Fig. 2C, D). LPS-induced TNF-α secretion in marmoset PCLS correlated significantly with LPS-induced TNF-α secretion in marmoset whole-blood cultures (Fig. 2E, r_s_  = 1.0, p  = 0.0004) and with LPS-induced TNF-α secretion in human PCLS (Fig. 2F, r_s_  = 0.9, p  = 0.01). Marmoset PCLS, however, showed a 50 times stronger TNF-α release to LPS than marmoset whole-blood cultures. No sex-specific differences in whole-blood cultures of female and male marmosets could be revealed (data not shown).

### Therapeutic Intervention with the Phosphodiesterase-4 Inhibitor Roflumilast Prevents LPS-induced TNF-α Release in Marmoset and Human PCLS

**Figure 3:**
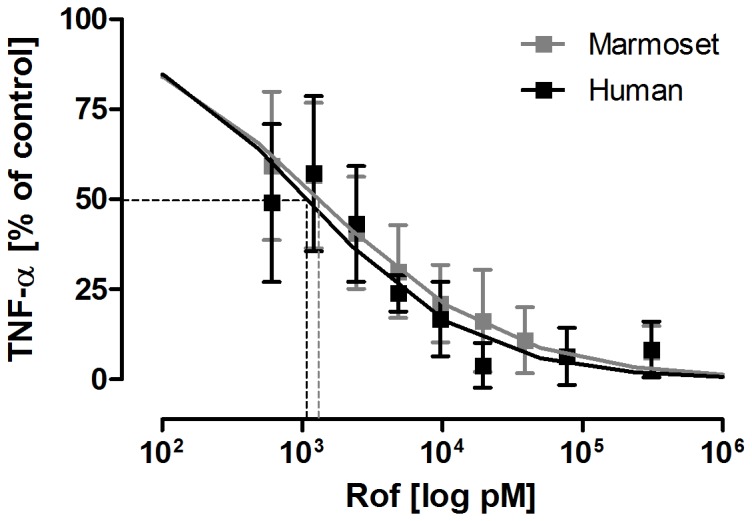
Dose-response curves of roflumilast in marmoset and human PCLS. Marmoset and human PCLS were incubated with 100 ng/mL LPS and increasing concentrations of roflumilast (rof, 0.6–310 nM) for 24 hours. The half maximal inhibitory concentrations were detected at 1.3 nM for marmoset and 1.1 nM for human (dashed lines). Data are presented as mean ± SEM, marmoset: n  = 5; human: n  = 4. TNF-α concentration in supernatants was determined by ELISA. Marmoset  =  grey symbols, human  =  black symbols.

Dose-response curves of the PDE4 inhibitor roflumilast were created for marmoset and human PCLS to assess analogies between both species as well as to estimate a dose for *in vivo* usage. Roflumilast efficiently suppressed LPS-induced TNF-α secretion in marmoset and human PCLS, revealing IC_50_ values of 1.3 nM (pIC_50_ = 8.88) and 1.1 nM (pIC_50_ = 8.97), respectively (Fig. 3). Based on these data, the animals received roflumilast in a dose of 7 µg/kg bw, which is comparable to the dose used in humans [12].

### Anti-inflammatory Treatment Reduces LPS-induced Changes in BAL Cell Composition

**Figure 4:**
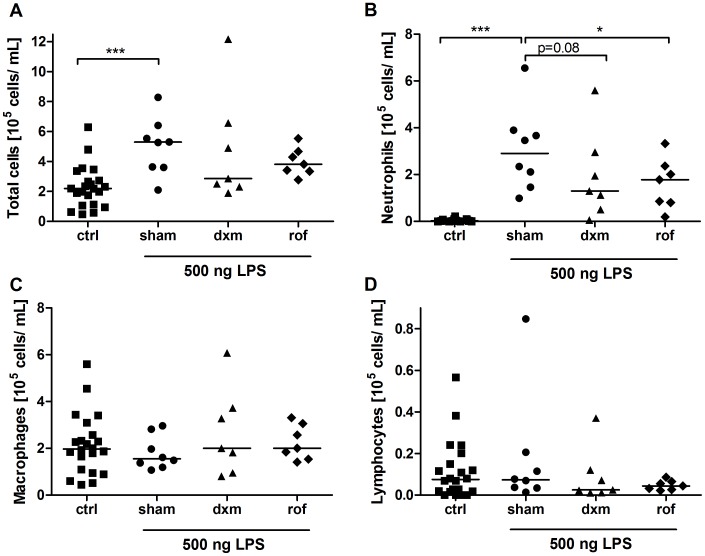
Changes in absolute cell numbers in bronchoalveolar lavage (BAL) fluid after LPS challenge. Sham-treated, dexamethasone (dxm)-treated, and roflumilast (rof)-treated marmosets were intrabronchially challenged with 500 ng LPS. Eighteen hours later, ipsilateral BAL was performed. Total cells (A), neutrophils (B), macrophages (C), and lymphocytes (D) were differentiated and quantified using light microscopy after Pappenheim staining. Data are presented as scatter dot plot with median, *p<0.05, ***p<0.001, one-tailed Mann-Whitney test against sham.

**Figure 5:**
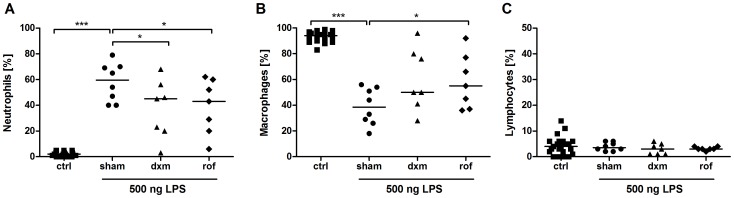
Changes in relative cell numbers in bronchoalveolar lavage (BAL) fluid after LPS challenge. Sham, dexamethason (dxm) and roflumilast (rof)-treated marmosets were intrabronchially challenged with 500 ng LPS. Eighteen hours later, an ipsilateral BAL was performed. Relative numbers of neutrophils (A), macrophages (B), and lymphocytes (C) were determined. Cells were quantified and differentiated using light microscopy after Pappenheim staining. Data are presented as scatter dot plot with median; *p<0.05, ***p<0.001, one-tailed Mann-Whitney test against sham.

Acute lung inflammation was induced by using an LPS dose of 500 ng, which was administered unilaterally by MicroSprayer® under bronchoscopic supervision. The study was performed in two cycles. The exact allocation of individual animals to treatment groups is shown in table 1. 9 out of 16 animals were used in both independent study cycles (Tab. 1). Altogether, two LPS challenges were found to be ineligible because of a BAL fluid recovery volume of less than 2 mL. In another LPS challenge bronchoscopy was not feasible because of an undersized throat (Tab. 1). Baseline lavages were performed before each study cycle revealing almost only macrophages (Fig. 4C, 5B, 6A). Therefore, results of both study cycles were pooled due to randomized character and identical performance of each cycle. Moreover, the observed effects were comparable between both cycles.

**Figure 6:**
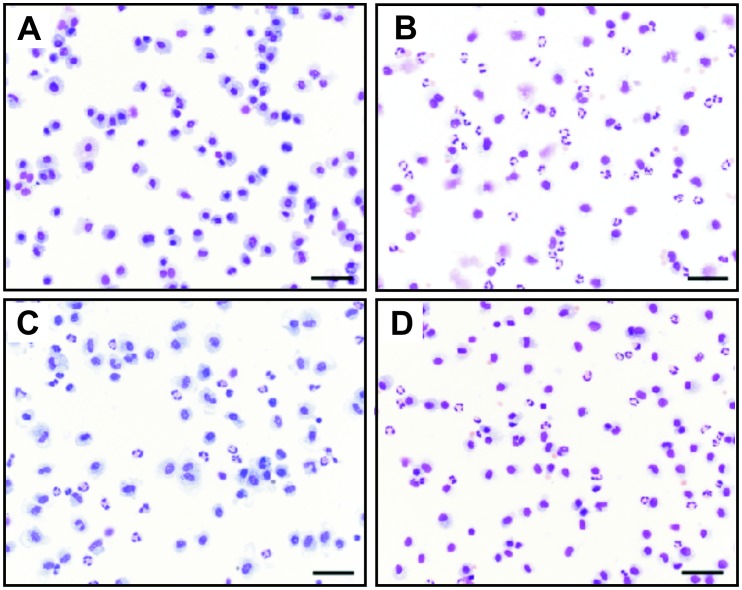
LPS-induced changes in bronchoalveolar lavage (BAL) fluid. Representative cytospots of BAL at x200 original magnification after Pappenheim staining. (A) Macrophages are the predominant cell type in unchallenged lung lobes. (B) LPS challenge induced strong neutrophilic influx in sham-treated animals. This effect could be significantly attenuated by (C) roflumilast and (D) dexamethasone pre-treatment. Scale bar  = 50 µm.

BAL was performed 18 hours after LPS challenge to monitor cellular changes and changes on cytokine levels. LPS induced a significant influx of inflammatory cells with increased total cell counts into the challenged lung lobe (Fig. 4A, p = 0.0007). Neutrophils showed the most pronounced increase after LPS challenge (Fig. 4B, 5A, 6B). In sham-treated animals (positive control) absolute macrophage (Fig. 4C, p = 0.26) and lymphocyte counts (Fig. 4D, p = 0.35) were not augmented. Relative macrophage counts, but not lymphocyte counts, were significantly decreased (Fig. 5B, p<0.0001, 5C). Eosinophils were not observed.

Compared with the positive control sham, pre-treatment with roflumilast resulted in a statistically significant decrease in absolute and relative neutrophil cell numbers in BAL (Fig. 4B, p = 0.04; Fig. 5A, p = 0.047, Fig. 6C). Moreover, relative but not absolute numbers of macrophages were significantly increased after roflumilast pre-treatment (Fig. 5B, p = 0.04; Fig. 4C, p = 0.09). Roflumilast pre-treatment had no influence on lymphocyte counts (Fig. 4D, 5C).

Pre-treatment with the treatment control dexamethasone revealed also a reduction in neutrophil numbers (Fig. 4B, Fig. 5A, Fig. 6D) compared to the positive control sham. Additionally, there was no effect on macrophages and lymphocytes after dexamethasone pre-treatment (Fig. 4C, p = 0.23; Fig. 4D, p = 0.1). All changes in BAL fluid were fully reversible after a minimum of three weeks post LPS challenge and returned to baseline levels within this time period (data not shown). Moreover, analysis of haemograms showed a significant increase in blood neutrophils in all individuals 18 hours after LPS-challenge compared to baseline levels, but no increase in blood TNF-α was detectable (data not shown). Blood neutrophilia was fully reversible within three weeks after LPS challenge. In line with pre-studies in whole-blood cultures of male and female marmosets, no sex-specific differences within the groups could be revealed.

### Anti-inflammatory Treatment Reduces LPS-induced Changes in Cytokines in BAL Fluid

**Figure 7:**
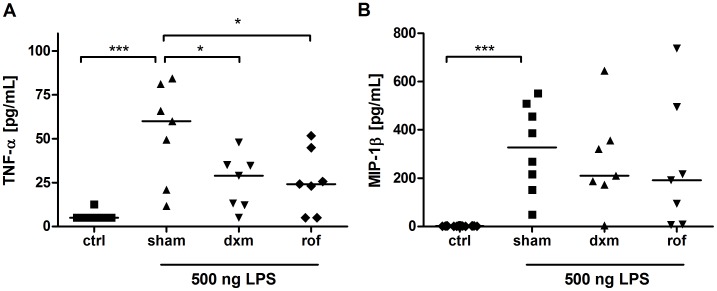
Analysis of bronchoalveolar lavage (BAL) fluid. TNF-α content in BAL fluid of marmoset monkeys was significantly reduced in dexamethasone-treated (dxm, p = 0.04) and roflumilast-treated (rof, p = 0.049) animals in contrast to sham-treated individuals (A). MIP-1β concentrations in BAL fluid were reduced by trend in both treatment groups (sham vs. dxm: p = 0.27; sham vs. rof: p = 0.17) (B). Data are presented as scatter dot plot with median, *p<0.05, ***p<0.0.01, one-tailed Mann-Whitney test against sham.

The pro-inflammatory cytokines TNF-α and MIP-1β were measured to analyze the efficacy of the different treatments. On average, TNF-α and MIP-1β levels in BAL fluid were increased 10- and 200-fold, respectively, after LPS challenge (Fig. 7A, B). Roflumilast (p = 0.048) and dexamethasone (p = 0.036) pre-treatment reduced TNF-α levels significantly. Furthermore, there was a tendency towards attenuation of MIP-1β secretion.

## Discussion

The present study aimed to establish a tiered LPS-induced acute lung inflammation model in marmoset monkeys. Two major aspects were addressed: firstly, the suitability of marmoset PCLS exhibiting features of LPS-induced acute lung inflammation was evaluated. Secondly, based on the preliminary studies in PCLS the feasibility of using a single unilateral LPS provocation and subsequent lavage in marmoset monkeys *in vivo* combined with successful anti-inflammatory treatment was described. This tiered testing strategy might improve the prediction of possible efficacy of newly developed experimental therapeutics in human clinical trials.

LPS-induced lung inflammation models are widely used as short-term models with airway and parenchymal changes as well as inflammatory cytokine up-regulation to test drugs against respiratory diseases including ALI and COPD [26]; [27]. Especially rodent models are commonly used in this context, but their readouts are often not predictive enough due to the absence of human-relevant molecular specifications, such as crucial discrepancies in receptor expression of immunity-related genes [18]. As a result, some pharmaceuticals are effective in rodents, but show no responses in humans, and *vice versa*. This was particularly fatal in the case of thalidomide, which was not teratogenic in rodents, but in NHP and in humans [28]. Therefore, alternatively to rodents further validation in phylogenetically most closely related animals is needed to translate findings to the human situation. So far, LPS-induced lung inflammation models have been established in cynomolgus macaques *(Macaca fascicularis*) [12]. This Old World monkey species is, however, more difficult to bread and to handle and thus very cost intensive. Contrary, the small New World monkey common marmoset is less demanding in this respect, and shows high homologies to humans, too [29]. Marmoset monkeys share 86% analogy in the amino acid residues of immunity related genes including IL-6 and IL-1β when compared to humans [18]. Additionally, marmoset monkeys exhibit strong analogies in anatomical and physiological aspects of the lung [25]; [30]. Marmoset monkeys are, therefore, a useful alternative to existing models in rodents and Old World primates for the development of novel drugs against human diseases. This is also reflected by the use of marmoset monkeys for toxicity testing. Canakinumab (Ilaris®_,_ Novartis Europharm Limited, Horsham, UK) a IL-1β blocker for example, was tested in marmoset monkeys due to a lack of cross-reactivity in rodents as well as in cynomolgus macaques [31]. Moreover, we recently demonstrated great analogies between marmoset and human lung tissue regarding mediator-induced bronchoconstriction [25]. Due to the urgent need of predictive animal models for human diseases, we investigated to what extent marmoset monkeys represent a suitable model of LPS-induced acute lung inflammation.

We could show a dose-dependent increase of TNF-α release in PCLS after LPS stimulation showing an EC_50_ of 22 ng/mL. This concentration was utilized to deduce the instillation dose of 500 ng LPS for the *in vivo* approach by taking into account that the half volume of an adult marmoset lung is 25 mL. Since LPS is a contaminant in cigarette smoke, smokers inhale approximately 500 ng LPS by smoking 3–4 cigarettes. Each cigarette contains 120±64 ng bioactive LPS in mainstream smoke, which results in an estimated delivered amount of 2.4 µg LPS by smoking one pack of cigarettes per day [32]. This LPS dose is similar to the level of LPS exposure of cotton textile workers suffering from adverse health effects [33]. Moreover, human clinical trials most commonly used even higher concentrations varying between 40 and 100 µg LPS applied via inhalation for investigating anti-inflammatory effects of certain drugs [34]–[36]. In this context, the estimation of the deposited LPS dose is, however, difficult due to the fact that a part of the LPS gets lost due to exhalation. Additionally, in chronic disease potentially pathogenic bacteria colonize the airways of patients producing also a small amount of endotoxin, which contributes to the progression of the disease since LPS is active at femtomole levels [37]. In particular, Sethi and colleagues assessed an amount of 36 mEU/mL endotoxin by using the limulus ameobocyte assay corresponding to approximately 3.6 pg/mL LPS which is thereby circa 6000-times lower than our instilled LPS dose [37]. In rodent models of ALI or ARDS even higher LPS doses have been used varying between 500 µg/kg bw injected intratracheally and 10 mg/mL LPS applied via inhalation to induce a profound lung inflammation [38]–[40].

Furthermore, we could reveal a high and significant correlation between marmoset whole-blood cultures and marmoset lung tissue regarding TNF-α release. The TNF-α level in WBA was, however, 50-fold lower compared to PCLS, suggesting a higher number of responding cells in marmoset lung tissue. Our experiments have clearly demonstrated high correlation of marmoset and human lung tissue regarding the distinct production of TNF-α in response to LPS. Similarly to human PCLS [7], the corticosteroid dexamethasone showed anti-inflammatory effects in marmoset monkey PCLS regarding TNF-α and MIP-1β secretion. Thus, the discussed steroid resistance due to decreased binding affinity of the glucocorticoid receptor in other New World monkey species, such as the squirrel monkey *(Samiri sciureus)*, is not an issue in the marmoset monkey [41]. The similarities in marmoset and human PCLS could further be confirmed by analogous pIC_50_ values (8.9 and 9.0, respectively) for the PDE4 inhibitor roflumilast. This is in line with previously shown pIC_50_ values of 7.7 in human WBA [42]. In fact, we previously used human PCLS as a physiologically relevant acute inflammation model showing high homology to the *in vivo* situation [7]. Because of the high analogies *ex vivo* [11]; [43], we decided to use the same dosage of roflumilast in our *in vivo* approach that was effective in human clinical trials.

In the present study, we performed a unilateral LPS challenge under bronchoscopic supervision in marmoset monkeys, followed by BAL 18 hours later. A similar approach has been used in human clinical trials. O’Grady *et al.* were the first to show that local endotoxin-induced lung inflammation in healthy human subjects results in strong neutrophilic influx and increase in inflammatory mediators 6 hours after provocation [44]. Most pro-inflammatory cytokines, however, returned to baseline levels after 24 hours [44]. Additionally, Hohlfeld *et al.* used segmental endotoxin instillation for assessment of the PDE4 inhibitor roflumilast [11]. For the purpose of the present study, a time period of 18 hours was used, in order to cover cellular changes in BAL fluid and cytokine up-regulation in lung tissue due to the fact, that especially neutrophils influx represents a hallmark of inflammation [11]; [45]. Both, TNF-α levels and numbers of neutrophils were significantly diminished in the roflumilast-treated animals as well as in the treatment-control dexamethasone. Thus, in marmoset monkeys with LPS-induced lung inflammation, roflumilast acts as an anti-inflammatory agent like in humans [11].

The acute LPS challenge was used to model TNF-α and MIP-1β increase as well as neutrophilia, which are hallmarks of complex respiratory diseases including ALI/ARDS and COPD. However, COPD represents a slowly progressive chronic respiratory disease implicating that only its pro-inflammatory features can be modeled in an acute approach. Additionally, COPD comprises airflow limitation and a variety of pathological changes in the peripheral airways and lung parenchyma such as chronic inflammation, tissue damage, emphysema resulting in airway fibrosis and alveolar destruction [45]. These features can only be mirrored in chronic studies which require LPS administration over several weeks. Appropriate studies have been performed in rodents and guinea pigs resulting in COPD-associated pathological changes such as enlarged air spaces, remodelled airways with thickened walls, and increased goblet cells [27]; [46]; [47].

The use of a relatively poor characterized NHP species is an important limitation of the present model, which becomes particularly obvious in the restricted scope of marmoset-specific analytics. Reliable analysis of alterations on cytokine level only comprises TNF-α and MIP-1β levels, contrary to the variety on murine and human commercially available tools. Cell differentiation with common cytology methods is, however, possible. Moreover, the use of NHP species for biomedical research is often viewed critical due to ethical concerns [48]. But the evidence of the scientific value of marmoset monkeys as a predictor for efficacy and adverse effects in humans due to the closer similarities to human metabolism and enzyme structures compared to rodents, dogs, and pigs is increasing [49]; [50]. Furthermore, the marmoset monkey exhibits similar pharmacodynamic effects as humans [51]. Marmoset monkeys might, therefore, help to clarify the scientific gap between commonly performed mouse models, human disease models, and human diseases [52].

In conclusion, LPS-induced acute lung inflammation in marmoset monkeys displays a promising model with good cost-benefit ratio for testing new drugs of human respiratory diseases. Especially highly specific anti-inflammatory pharmaceuticals targeting pro-inflammatory cytokine secretion as well as neutrophilic influx might be tested in this model. Additionally, PCLS are a useful tool to estimate a dose range for *in vivo* usage. Thus, tiered testing in marmoset monkeys can be applied for the examination of potential new therapeutics.
